# The ocean's role in polar climate change: asymmetric Arctic and Antarctic responses to greenhouse gas and ozone forcing

**DOI:** 10.1098/rsta.2013.0040

**Published:** 2014-07-13

**Authors:** John Marshall, Kyle C. Armour, Jeffery R. Scott, Yavor Kostov, Ute Hausmann, David Ferreira, Theodore G. Shepherd, Cecilia M. Bitz

**Affiliations:** 1 Department of Earth, Atmospheric and Planetary Sciences, Massachusetts Institute of Technology, 77 Massachusetts Avenue, Cambridge, MA 02139, USA; 2 Department of Meteorology, University of Reading, Reading, Berkshire, UK; 3 Department of Atmospheric Sciences, University of Washington, Seattle, WA, USA

**Keywords:** Southern Ocean, ozone hole, greenhouse gas forcing, climate model

## Abstract

In recent decades, the Arctic has been warming and sea ice disappearing. By contrast, the Southern Ocean around Antarctica has been (mainly) cooling and sea-ice extent growing. We argue here that interhemispheric asymmetries in the mean ocean circulation, with sinking in the northern North Atlantic and upwelling around Antarctica, strongly influence the sea-surface temperature (SST) response to anthropogenic greenhouse gas (GHG) forcing, accelerating warming in the Arctic while delaying it in the Antarctic. Furthermore, while the amplitude of GHG forcing has been similar at the poles, significant ozone depletion only occurs over Antarctica. We suggest that the initial response of SST around Antarctica to ozone depletion is one of cooling and only later adds to the GHG-induced warming trend as upwelling of sub-surface warm water associated with stronger surface westerlies impacts surface properties. We organize our discussion around ‘climate response functions’ (CRFs), i.e. the response of the climate to ‘step’ changes in anthropogenic forcing in which GHG and/or ozone-hole forcing is abruptly turned on and the transient response of the climate revealed and studied. Convolutions of known or postulated GHG and ozone-hole forcing functions with their respective CRFs then yield the transient forced SST response (implied by linear response theory), providing a context for discussion of the differing warming/cooling trends in the Arctic and Antarctic. We speculate that the period through which we are now passing may be one in which the delayed warming of SST associated with GHG forcing around Antarctica is largely cancelled by the cooling effects associated with the ozone hole. By mid-century, however, ozone-hole effects may instead be adding to GHG warming around Antarctica but with diminished amplitude as the ozone hole heals. The Arctic, meanwhile, responding to GHG forcing but in a manner amplified by ocean heat transport, may continue to warm at an accelerating rate.

## 1. Introduction

**Figure 1.**
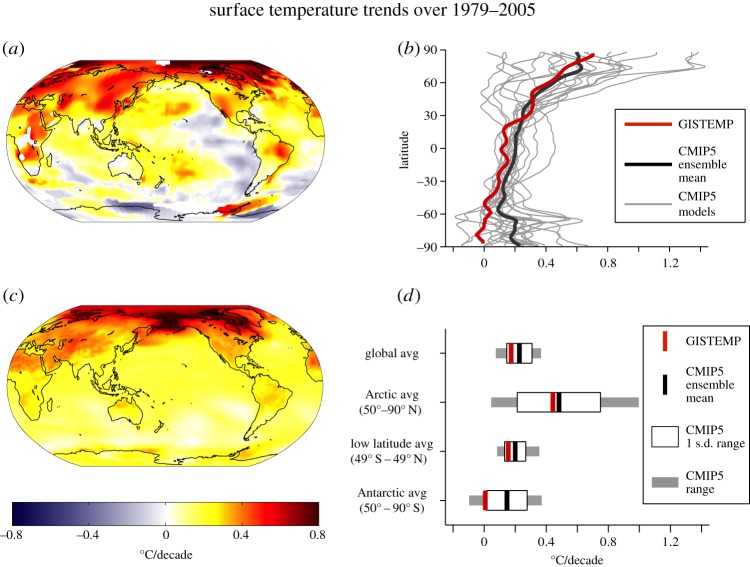
Surface temperature trends over 1979–2005 from (*a*) the GISS Surface Temperature Analysis (GISTEMP) [1], (*c*) an ensemble of CMIP5 models. (*b*) Zonal-mean surface temperature trend from GISTEMP (red line), CMIP5 ensemble mean (black line), individual CMIP5 models (grey lines). (*d*) Surface temperature trends averaged over latitude bands (global, Arctic, low-to-mid latitudes and Antarctic), for GISTEMP (red line) and CMIP5 ensemble mean (black line); the white boxes show the CMIP5 ensemble ±1 s.d. range, and the grey boxes show the full CMIP5 ensemble range. All trends are expressed in °C/decade.

Over the last few decades, the two polar regions of our planet have exhibited strikingly different behaviours, as is evident in observed decadal trends in surface air temperature shown in figure 1. The Arctic has warmed, much more than in the global average, primarily in winter, while Arctic sea-ice extent has decreased dramatically [2]. By contrast, the eastern Antarctic and Antarctic plateau have cooled, primarily in summer, with warming over the Antarctic Peninsula and Patagonia [3,4]. Moreover, sea-ice extent around Antarctica has modestly increased [2].

Observed and modelled surface temperature trends (in °C/decade) during the 1979–2005 period are shown in figure 1, together with quantification of the observed and modelled standard deviation. It is clear that there are very different observed Arctic and Antarctic temperature trends and each differs from the global trend with the Arctic warming more than the Antarctic. Moreover, there is significant spread between models: the ensemble-mean tends to be positively biased in the Antarctic, and the model spread is particularly large in the Arctic. The annually averaged temperature in the Arctic has increased by over twice that of the global mean (a phenomenon known as Arctic amplification). Since 1979, the beginning of the reliable satellite record, Arctic summer sea-ice extent has decreased by order 12% per decade, with smaller reductions in winter. Coupled models suggest that under greenhouse gas (GHG)-induced warming, the Arctic will warm the most: models generally exhibit enhanced warming and sea-ice loss in the Arctic in response to increasing GHGs, but the observed changes over the past decade lie at the upper limit of the model projections [5,6]. According to projections of the Fifth Assessment Report of the Intergovernmental Panel on Climate Change [7], by the end of the twenty-first century, the annual average surface air temperature in the Arctic will increase by 5.2–11.4°C (5–95% confidence range) under ‘business-as-usual’ (RCP8.5) emissions. Decreases in sea-ice extent and thickness are projected to continue, and some models suggest that the Arctic Ocean will be free of sea ice in late summer by mid-century (as discussed in [8]). By contrast, under the same emission scenario, the Antarctic is projected to warm by 1.1–5.1°C over the twenty-first century.

Many mechanisms are at work in ‘Arctic amplification’ (see, e.g., the overview of [9] and references therein). A positive snow and sea-ice albedo feedback plays a significant role in amplifying the warming signal [10]. The albedo feedback operates in summer when solar radiation is maximal. Where sea ice is lost and water is exposed, warming due to absorbed shortwave radiation can be large and enhance sea-ice loss through lateral melt [11]. In addition to these processes, the warmed ocean mixed layer delays sea-ice growth [12], and thus influences wintertime surface temperatures through a thinner ice pack. Because the Arctic atmosphere is stably stratified by thermal inversion at the surface, any warming that occurs there does not reach far up into the troposphere. Moreover, the surface energy balance is very sensitive to processes going on in the planetary boundary layer and cloud radiative processes (e.g. [13]). Additionally, as is emphasized in the work presented here, the climate of the polar caps is determined by more than regional and vertical energy balance, as lateral advection of heat by atmosphere and ocean circulation also plays a significant role.

The area poleward of the 70° N latitude circle receives more energy due to atmospheric transport than it does from the Sun. Moreover, this lateral heat-flux convergence is largely balanced by outgoing infrared radiation, with surface fluxes contributing a relatively small amount to the energy budget [14]. The sensitivity of poleward atmospheric heat transports to climate change is currently under debate (e.g. [15]): polar amplification reduces meridional temperature gradients, which might be expected to reduce meridional atmospheric heat transport from lower latitudes, thus counteracting a portion of the amplification. Some studies argue that anomalous atmospheric heat transport, mainly due to increased moisture, have given rise to greater atmospheric warming above the surface of the Arctic [16,17]. However, the validity of the analysed atmospheric trends on which such studies are based is disputed [18–20]. Beyond atmospheric heat transports, the high-latitude response to greenhouse forcing may involve anomalous ocean heat transport into the Arctic; as we shall see, this occurs even if a weakened meridional overturning circulation (MOC) diminishes the heat transport at lower latitudes [10,21]. In addition, the ocean can act as a reservoir for the heat gained in summer while the sea ice retreats, storing it through winter months [12].

The mix of ongoing processes in the Antarctic is rather different from those in the Arctic. The dramatic depletion of the Antarctic ozone since the late 1970s has introduced a major perturbation to the radiative balance of the stratosphere with a wide range of consequences for climate. There is strong evidence that ozone loss has significantly altered the climate of the Southern Hemisphere troposphere, including the surface, with implications for ocean circulation, the cryosphere and coupled carbon cycle [22]. Observations indicate a poleward shift of the Southern Hemisphere atmospheric circulation over the past few decades, predominantly in late spring and summer. This shift has been attributed to polar ozone depletion in the Antarctic lower stratosphere [3,4]. The observed changes have the structural form of the Southern Annular Mode (SAM) in its positive phase: the surface wind maximum, the storm tracks, and the edge of the Hadley cell all shift poleward. While similar changes, with the same sign, have been reproduced in models under GHG warming scenarios (e.g. [23,24]) they are also found in response to imposed ozone depletion (e.g. [25–28]). In fact, on the basis of GCM studies in which both forcings were included, separately and together, it is believed that ozone depletion has been the primary cause of the observed wind changes [29,30]. In the future, assuming ozone depletion weakens as expected, the effects of GHG and ozone forcings may no longer act in the same sense on surface winds.

Changes in the Southern Hemisphere westerlies (and SAM) have been linked to changes in sea-surface temperatures (SSTs) and sea-ice extent around Antarctica on interannual time scales (e.g. [4,31–35]). A positive SAM induces an overall transient cooling through the enhanced Ekman transport of cold surface waters northward from Antarctica promoting sea-ice growth. There is, however, debate about the cause of the observed decadal trends in sea-ice extent, which show a small net expansion around Antarctica but large regional trends of opposing sign. Coupled models suggest that initial (interannual) cooling around Antarctica induced by a positive SAM reverses to one of warming as time proceeds [36–39]. The warming tendency and sea-ice retreat is a consequence of enhanced upwelling of warm water from depth around Antarctica associated with strengthening westerly winds. Natural variability may also be playing a role in the observed signals [40,41], even if trends in the SAM itself were to be absent.

The links between the upwelling of deep water in the Southern Ocean (SO) and the Southern Hemisphere westerly winds and consequences for climate have long been an area of active research (e.g. [42]). Although changes in the slope of density surfaces in the Antarctic Circumpolar Current (ACC) cannot yet be detected [43], ocean observations indicate a freshening of Antarctic Intermediate Water [44,45] and a substantial warming of the SO equatorward of the ACC at all depths [46,47] which may be linked to atmospheric forcing [48]. Modelling studies and theory, however, suggest that eddy transport in the ACC can partially compensate for changes in Ekman transport ameliorating changes in the strength of the MOC [49–51].

Enhanced communication of the interior ocean with the surface could have marked effects on the Earth's climate through changes in rates of heat and carbon sequestration as well as consequences for ice shelves around Antarctica which may be vulnerable to enhanced upwelling of warm water from depth [52–56]. The stratification of the SO is also delicately poised and sensitive to changes in the freshwater balance [57,44].

These introductory remarks make clear that many competing effects are at work in modulating the response of polar climates to anthropogenic forcing. The main goal of this paper is to set out a framework for thinking about and quantifying differing responses of the Arctic and Antarctic to anthropogenic forcing. We organize our discussion around ‘climate response functions’ (CRFs), i.e. the response of the climate to ‘step’ changes in anthropogenic forcing in which GHG and/or ozone-hole forcing is abruptly turned on and the transient response of the climate revealed and studied. As discussed in [58], for example, step function response experiments have a long history in climate science and are related to ‘impulse’ (Green's) function responses. Here, we will use linear response theory to probe the role of the ocean in shaping the asymmetric response of polar climates to anthropogenic forcing, and in so doing attempt to expose the elemental processes at work. We will study responses to a step-change in thermal forcing and a step-change in mechanical (wind) forcing.

In §2, we consider CRFs associated with GHG forcing and then, in §3, CRFs associated with wind changes induced by ozone-hole forcing. In §4, we convolve time histories and projections of GHG and ozone-hole forcing with these CRFs to contrast the response of the high-latitude climate. In §5, we summarize and conclude.

## 2. Modulation of the sea-surface response to greenhouse gas forcing by ocean circulation

### (a) Asymmetric response of Arctic and Antarctic surface climates to greenhouse gas forcing

**Figure 2.**
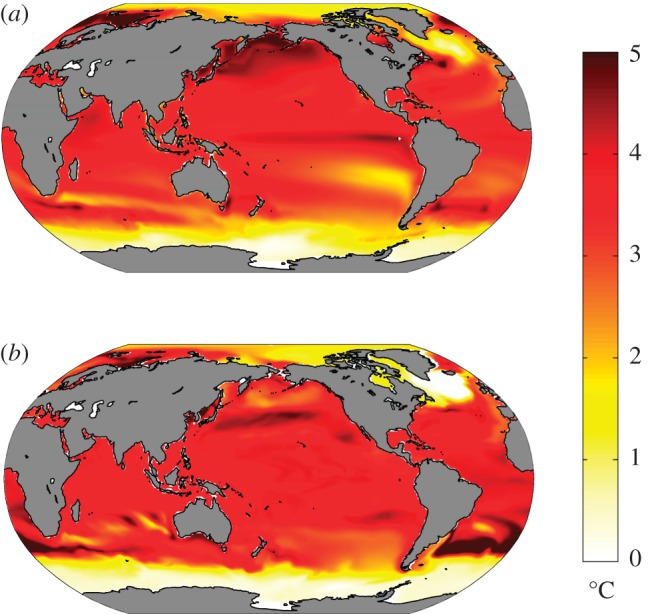
(*a*) Ensemble-average SST anomalies 100 years after an abrupt quadrupling of CO_2_ in 15 CMIP5 models. (*b*) SST anomalies after 100 years of an ocean only configuration of the MITgcm induced by a uniform downwelling flux of 4 W m^−2^ and damped at a uniform rate of 1 W m^−2^ K^−1^, as described in §2.

**Figure 3.**
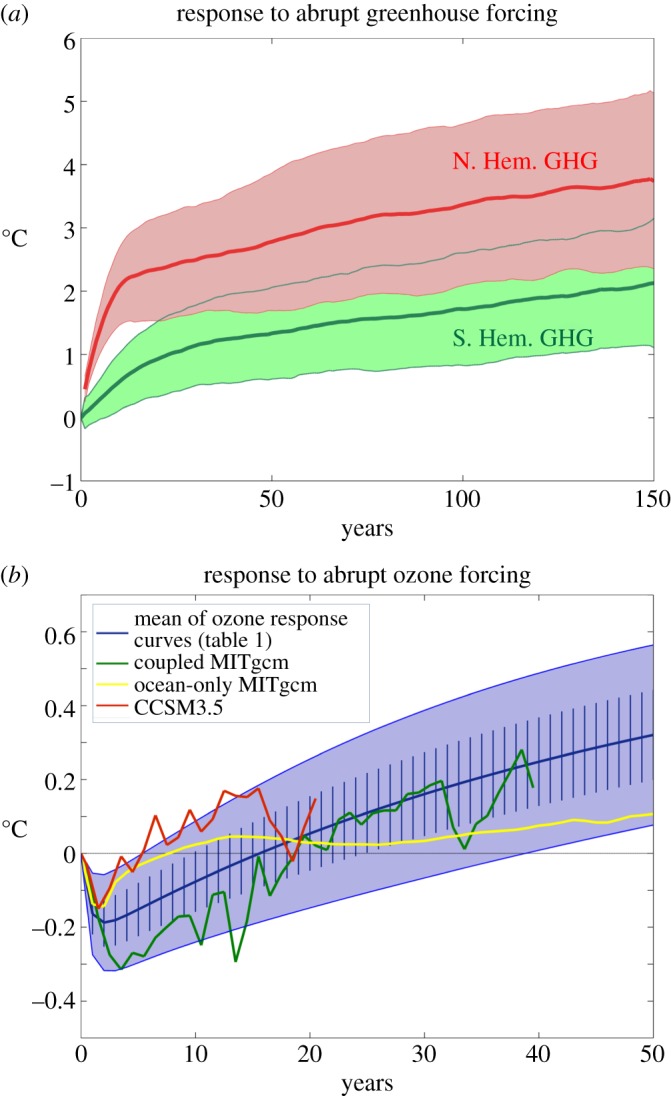
Sea-surface temperature CRFs for: (*a*) GHG forcing computed from an ensemble of 15 CMIP5 models under quadrupling of CO_2_. The Arctic is defined as north of 50° N (in red) and the Antarctic between 50° S and 70° S (in green). Thick lines denote the ensemble mean and the shaded area spans 1 s.d.; (*b*) ozone-hole CRFs based on the analytical expression equation (4.2) appropriate for a repeating annual cycle of amplitude approximately 100 DU. The thick blue line is the ensemble mean of the analytical response curves (table 1). Vertical hash marks represent 1 s.d., and the solid blue shading spans two standard deviations. The green curve is the ensemble mean SST response between 50° S and 70° S from abrupt ozone depletion experiments with the coupled MITgcm. The red curve shows the ensemble-mean evolution of the ‘cold pole’ of the SST dipole induced by abrupt ozone-hole forcing in the CCSM3.5 NCAR model from [39]. The yellow curve is the SST response between 50° S and 70° S from an abrupt SAM perturbation experiment with the ocean-only version of the MITgcm described in §3.

Coupled climate models suggest that under GHG forcing, the Arctic may be expected to warm more rapidly than the Antarctic. For example, figure 2*a* shows the ensemble-average response of SST after 100 years in CO_2_ quadrupling experiments computed from 15 general circulation models participating in the Coupled Model Intercomparison Projects phase 5 (CMIP5, [59]). In such experiments, coupled models were integrated out to (quasi-) equilibrium forced with pre-industrial GHG concentrations. The CO_2_ concentration was then abruptly quadrupled to study how the coupled climate evolved towards a new equilibrium. The evolution of the coupled system is strongly modulated by the sequestering of heat into the ocean's interior, as has long been discussed in the literature—see, e.g., [60] and references therein. Note that SST does not warm up uniformly but instead there is a rich spatial structure in its response after 100 years. In some regions of the globe, SST increases by more than 4°C, whereas in others, particularly in the circumpolar band around Antarctica, SST increases by less than 1°C. Marked hemispheric and polar asymmetries are evident with SSTs in the Northern Hemisphere being generally considerably warmer than in the Southern Hemisphere. Figure 3*a* illustrates the time evolution of SST as a function of latitude by zonally averaging over Arctic and Antarctic bands.^1^ These are our GHG CRFs. Delayed warming is evident in the SO around Antarctica; Arctic amplification is clearly present with SSTs rising much more rapidly than around Antarctica.

What is the essential ‘physics’ behind the amplitude and timing of such differing polar responses to GHG forcing? Are they a consequence of the interaction between GHG forcing and local radiative feedback processes (e.g. due to cloud changes) that perturb the energy budget in differing ways over the two poles? Are they driven by different responses in atmospheric circulations and energy transports? Do they reflect different patterns of storage of anthropogenically induced temperature signals in the deep ocean? Here, we suggest that, independent of the above mechanisms, the patterns and timing of warming evident in figures 1–3 can be largely explained in terms of the advection of anthropogenic temperature anomalies by the background ocean circulation.

### (b) Role of ocean circulation

To isolate the role of ocean circulation and expose some of the essential processes at play in setting the patterns in figure 2*a*, we take away details of the atmospheric component of the coupled system by running an ocean-only model based on the MITgcm [61,62] driven by an atmosphere that is represented in a highly parametrized, schematic way. The ocean model is typical of those used in contemporary coupled climate models. It is configured with realistic topography at 1° resolution with 50 vertical levels, and forced with analysed fields in a perpetual year. A hybrid latitude–longitude and cubed sphere configuration is used. The mesoscale eddy field is parametrized with an eddy diffusivity parameter set equal to a constant 850 m^2^ s^−1^, and mixed-layer processes are parametrized through the turbulent closure scheme described in [63]. Background diapycnal mixing is set to 10^−5^ m^2^ s^−1^. Precise details of the methodology are described in [64].

In our climate change experiments:

(1) We spin up a global version of the ocean model from the World Ocean Atlas (due to [65] which includes an Arctic analysis) for 300 years, using ‘normal year’ forcing [66] to compute, via bulk formulae, air-sea heat, freshwater and momentum fluxes. The run is continued for 10 additional years and all fluxes into the top of the ocean (including below the prognostic sea ice) are diagnosed daily; a 10-year mean of ‘daily forcing’ and SSTs is computed and stored as ‘data’. The reference solution is then continued on, driven by a repeating annual cycle of this daily ‘climatological’ surface boundary condition.
(2) The effect of warming due to GHG forcing is parametrized by imposing a spatially uniform and constant-in-time surface downwelling flux of 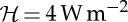. The anomalous flux is only applied to the ice-free ocean which therefore warms so that its *T* is typically greater than that of the reference solution *T*_ref_. We will call the difference ‘anthropogenic temperature’: *T*_anthro_=*T*−*T*_ref_. Likewise, the anthropogenic sea-surface temperature is given by SST_anthro_=SST−SST_ref_. Note that the wind field and the freshwater fluxes are not perturbed and the role of sea ice is considered passive, all of which are considerable simplifications.
(3) Climate ‘feedbacks’ are parametrized by introducing a damping term, −λSST_anthro_, where λ is a ‘climate feedback parameter’ chosen to be spatially uniform and have a value of 1 W m^−2^K^−1^.

One can question the simplicity and validity of these assumptions but, in this context, they can be turned to our advantage. In particular, because 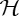 and λ are constant in space and time, any spatial patterns that emerge in the resulting temperature perturbations must be directly caused by, and hence attributable to, ocean circulation.

Figure 2*b* shows the SST perturbations after 100 years and can be directly compared to figure 2*a* from the ensemble of coupled climate models. Not only are gross patterns of the coupled model response captured in our ocean-only calculation, but also subtle details. Indeed, the similarity in spatial patterns is so striking that it tells us that they are largely a consequence of the underlying ocean circulation rather than (the much more complex and uncertain) processes occurring in the atmosphere under global change. CRFs from the ocean-only model (not shown here but discussed in [64]) also closely resemble figure 3*a*.

**Figure 4.**
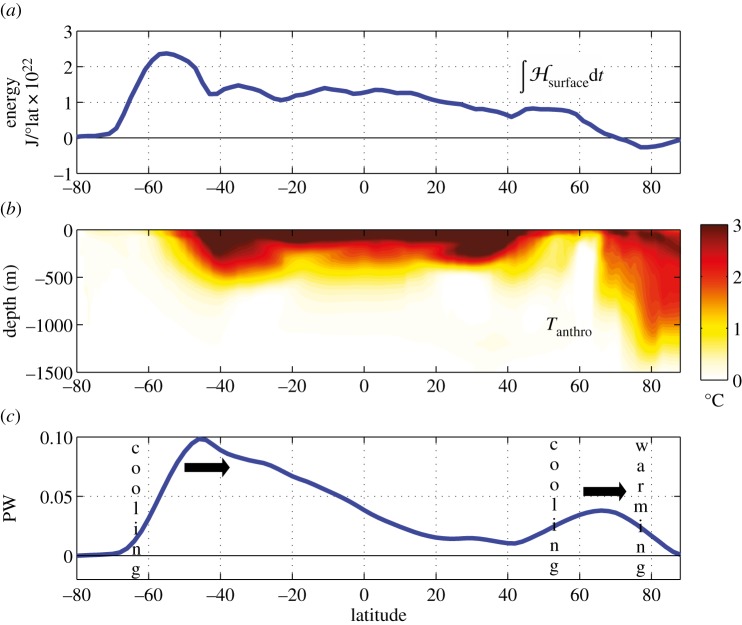
(*a*) Surface energy accumulation integrated over 100 years in J/°lat ×10^22^. (*b*) Meridional section of zonal-average *T*_anthro_ after 100 years from the ocean-only configuration of MITgcm whose SST_anthro_ is shown in figure 2*b*. (*c*) Anomaly in meridional ocean heat transport (in PW) after 100 years relative to a control integration. Latitudinal bands of implied ocean warming and cooling are marked.

A glimpse at the interior structure of the anthropogenic temperature signal (*T*_anthro_) is given in figure 4*b* where the zonal-average temperature perturbation is plotted. There is a clear interhemispheric asymmetry with *T*_anthro_ being much larger in the Arctic than in the Antarctic. The time-integrated anomalous air-sea fluxes over 100 years (energy accumulation) is plotted in figure 4*a* and reveals that most of the energy is fluxed into the ocean around Antarctica due to the delayed warming there. However, it is not stored around Antarctica. Instead, as can be seen in figure 4*c*, there is anomalous ocean heat transport northward away from Antarctica keeping its waters cool. The reverse is true in the Arctic. We see that the ocean carries heat into the Arctic (figure 4*c*), increasing its temperature to such an extent that heat is actually lost to the atmosphere over the Arctic (figure 4*a*).

The advective process shaping the response is largely associated with the upper cell of the ocean's meridional circulation with sinking in northern polar regions and upwelling in the SO around Antarctica (see the review [67]). This cell is a major interhemispheric asymmetry of the global climate, a consequence of differing hemispheric geometrical constraints on ocean circulation.

As discussed in [64], in the SO *T*_anthro_ evolves very much like a passive tracer, ‘injected’ at the sea surface, weakly damped at the surface by climate feedbacks but governed by an advection–diffusion equation in the interior. Here, to a good approximation, *T*_anthro_ remains sufficiently ‘small’ that even after 100 years or so it does not significantly affect ocean currents. However, this does not hold true in the North Atlantic, where changes in ocean currents induced by *T*_anthro_ (and the Atlantic Meridional Overturning Circulation—AMOC) contribute significantly to changes in ocean heat transport.

Before proceeding to a discussion of ozone hole impacts, important caveats should be mentioned. Our ocean-only strategy permits sea ice a role in establishing the mean stratification, but not in stratification changes. Moreover, changes in freshwater surface fluxes are not allowed. Changes in precipitation, ice sheet/shelf runoff, and sea-ice freshwater exchanges may all play a significant role under GHG forcing. Our calculations suggest, however, that the warming signal induced by anthropogenic GHG forcing is shaped by ocean circulation, with sea ice and freshwater effects playing a secondary role (cf., e.g., figure 2*a* and *b*).

## 3. Response of the Antarctic to ozone-hole forcing

What, then, is the effect of the ozone hole on the surface climate around Antarctica? The direct effect of ozone-hole forcing on the ocean's surface is essentially mechanical through its projection on to the surface winds associated with SAM (and thence air-sea heat and freshwater fluxes). This should be contrasted with GHG effects considered in §2 which are primarily manifested through thermodynamic processes.^2^ To further explore effects of anomalous winds we use the same ocean model described in §2 but now instead of perturbing it with a downwelling flux mimicking GHG warming, we perturb it through an anomaly in the wind field around Antarctica mimicking ozone-hole forcing. The procedure is as follows.

**Figure 5.**
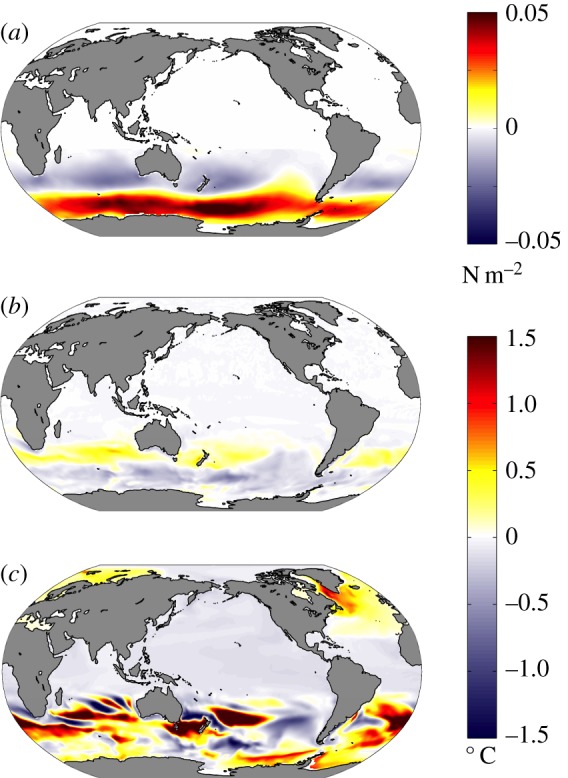
(*a*) Zonal wind-stress anomaly (in N m^−2^) applied to the ocean-only MITgcm, computed from the monthly 1980 to present NCEP-GODAS climatology representative of a +1 s.d. of the SAM obtained after linearly detrending both. (*b*) SST anomalies in °C after 1 year and (*c*) 50 years the ocean-only MITgcm simulation induced by anomalous SAM wind forcing shown in (*a*), as described in §3. Red indicates warming and blue cooling.

(1) We take the model described in point 1, §2 and perturb the forcing via the SAM wind stress field shown in figure 5*a*. This fixed pattern is multiplied by a spatially uniform factor that varies at one cycle per year peaking at the end of November, scaled so that the annual-mean wind-stress anomaly corresponds to the 1*σ* SAM pattern plotted in figure 5*a*. The resulting anomaly in the wind stress field is then added to the ‘stored’ daily forcing data over ice-free oceans. This crudely represents the forcing of SAM by the ozone hole which peaks in the summertime. Note that only the wind stress is perturbed and here we do not attempt to represent the effect of wind anomalies on air-sea latent and sensible heat fluxes.
(2) Climate feedbacks are again parametrized as described in point 3, §2.

The above procedure sidesteps complex issues that concern how and to what extent ozone-hole forcing projects onto SAM. Nevertheless, when we interpret our results in §4, it will be assumed that trends in SAM over the past few decades are primarily due to ozone-hole forcing, as argued for example in [29,30].

The SST_anthro_ field after 1 and 50 years of anomalous SAM wind-stress forcing is shown in figure 5*b*,*c*. Initially, we see a broadly axi-symmetric, dipolar SST anomaly pattern with cooling around Antarctica and warming further north. As noted earlier, this can readily be understood as the direct response of SST to anomalous advection by Ekman currents induced by (positive) SAM forcing. However, over time, widespread subsurface warming (in the top few hundred metres) of the ocean appears, which ultimately impacts surface temperatures. This is revealed in the evolution of the SST index obtained by averaging between 50° S and 70° S and plotted as a function of time in figure 3*b* (yellow line). Initially, we see a cooling and then a prolonged warming trend. Two timescales are at work: a ‘fast’ cooling period (several years) followed by a ‘slow’ warming trend (over decades), as discussed in detail in [39].

Also plotted in figure 3*b* are ozone-hole CRFs from two coupled models discussed in [39]—coupled MITgcm (green line) and CCSM3.5 (red line). These were obtained by introducing an ozone hole with a repeating annual cycle in these coupled models and ensemble-averaging to obtain the forced response. We see that in both models the initial response is one of cooling followed by a warming trend. However, the time to the crossover from cooling to warming is rather different and occurs more rapidly in CCSM3.5 than in MITgcm, as discussed in detail in [39]. Nevertheless, both coupled models, together with our ocean-only model, exhibit a two-time-scale response with an initial rapid cooling followed by a slower warming.

**Figure 6.**
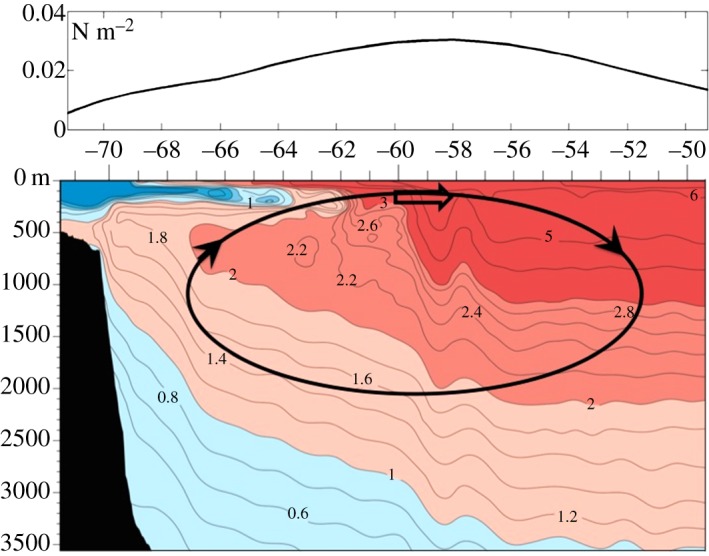
Meridional hydrographic section of temperature (WOCE section P19) stretching up to Antarctica on the left. The longitude range of the section is 85W–90W. The region of seasonal sea ice is coincident with cold water (blue tongue) at the surface overlying warmer water (red) below. Superimposed is the sense of the anomalous meridional overturning circulation associated with a positive SAM anomaly, with upwelling around Antarctica and downwelling further equatorward. This acts to warm the ocean just beneath the surface layer. The black line in the top panel shows the SAM-induced zonal wind-stress anomaly plotted in figure 5*a*, but at the longitude of the P19 section.

The mechanism of the warming trend involves the response of the interior ocean to SAM forcing. As sketched in figure 6, when the summertime SAM is in its positive phase, upwelling is induced around Antarctica with downwelling further north. In the region of upwelling, there is a temperature inversion (the surface is colder than waters below), a consequence of the melting/freezing and export of ice and resulting freshening of the surface waters. Thus, upwelling in response to SAM brings warm water up towards the surface in the band of seasonal sea ice. In the region of downwelling to the north, away from the region of seasonal ice, warm water is pushed down from the surface. Thus, in response to a positive SAM forcing, we expect to see, and indeed observed in figure 5*c*, widespread warming of the ocean just below the mixed layer. Over time, this warming signal becomes entrained into the mixed layer leading to a warming of SST.

As discussed in [39], the subsurface warming trend is governed by 
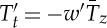
 where *w*^′^ is the anomalous upwelling induced by SAM forcing acting on the mean stratification 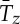 and the overbar is an average over the seasonal cycle: typically *w*^′^ and 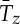 have opposite sign leading to a widespread warming trend.

Key processes that need to be understood and modelled include:

— how the ocean's overturning circulation responds to abrupt wind forcing as a function of timescale—see, for example, [51,68–71]. The Ekman response to a change in the wind is essentially instantaneous, but eddy contributions to the residual overturning circulation may become increasingly important as time progresses resulting in partial compensation of the Ekman response. These processes are crudely parametrized in and/or resolved in models and it is not at all clear that they can adequately capture the heat budget of the mixed layer which involve the parametrization of both skew and residual mesoscale eddy fluxes.
— the establishment of the near-surface stratification of the ocean in the region of seasonal sea ice. The stratification under ice is typically delicately balanced with both temperature and salinity playing a role [57]. This is very challenging to observe and capture in models.
— the spatial and temporal patterns of response that characterize not just the relationship between ozone-hole forcing and surface winds but also the effect of surface winds on SST and sea-ice cover. The ozone-hole CRF as defined here integrates over this detail, but local effects may be central to setting the regional patterns of response [35].
— the rate at which SST anomalies created by SAM are damped by air–sea interaction processes, as discussed in [39].

## 4. The combined effect of greenhouse gas and ozone-hole forcing on polar climates

If one knows the form of the GHG and ozone-hole CRFs and the respective GHG and ozone-hole forcing functions, convolving one with the other yields the predicted response. We present such calculations here for plausible CRFs and forcing functions and contrast the evolution of SST over the Arctic relative to the Antarctic.

More precisely we may write, following [58]:
4.1
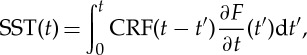

where *F* is the prescribed forcing function (in *W* m^−2^ for GHG forcing or Dobson units (DU) for ozone forcing), CRF is the step response function per unit forcing and SST is the response.

In §§2 and 3, we have discussed the contrasting forms of CRFs for GHG and ozone-hole forcing for the Antarctic and Arctic. Examples are plotted in figure 3 from a range of models. It is useful to express them as the sum of two exponential functions corresponding to a ‘fast’ and ‘slow’ process thus:
4.2
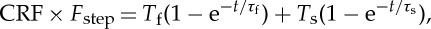

where the scaling factor *F*_step_ is the magnitude of the step function in the forcing *F* used to construct the CRF. The coefficients *T*_f_, *T*_s_, *τ*_*f*_ and *τ*_s_ depend on whether GHG or ozone-hole forcing is being considered, and whether we are in the Arctic or Antarctic, as set out in table 1. The GHG coefficients are estimated by fitting equation (4.2) to the curves shown in figure 3*a* obtained from quadrupling CO_2_ experiments. The ozone-hole coefficients are chosen to encompass the ensemble-average spread of the CRFs reported in [39] induced by a repeating annual cycle in ozone forcing of order 100 DU in two coupled models (the blue and red curves in figure 3*b*) and the CRF from the ocean-only SAM experiment described in §3 (the yellow curve). There are considerable uncertainties in all of these parameters, particularly those associated with the ozone-hole forcing. The blue shading in figure 3*b* indicates the spread in the family of curves computed from equation (4.2) with the parameters set out in table 1. Note that a critical difference between the ozone hole and GHG CRFs is that *T*^ozone^_f_ is negative, whereas 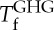 is positive: ozone-hole forcing promotes cooling of SST around Antarctica on fast timescales, whereas GHG forcing promotes warming. Note *T*^ozone^_s_ is positive because on long timescales the effect of ozone-hole forcing is a warming of the surface climate, as discussed in §3 and clearly evident in figure 5.

**Table 1.** Timescales (in years) and amplitudes (in K) of GHG and ozone-hole CRFs, equation (4.2), in the region indicated. The range of CRF curves implied by the tabulated parameters are indicated by the blue shading in figure 3*b* with the thick blue line indicating the centre parameters.

forcing	region	*T*_f_(K)	*T*_s_(K)	*τ*_f_(y)	*τ*_s_(y)
GHG	Arctic	2.2±0.7	5.5±0.7	4.5±0.5	520±200
GHG	Antarctic	1.0±0.6	7.0±0.5	12±3.0	900±200
O_3_	Antarctic	−0.26±0.09	1.08±0.07	0.66±0.24	54±21

As discussed in detail in [39], there is a large uncertainty in the processes that set the time-scale of the cross-over from cooling to warming in figure 3*b*. It would be desirable if similar ozone CRFs were performed with a range of coupled models. Such calculations, however, have yet to be carried out. In table 1, therefore, and as plotted in figure 3, we consider a rather wide range of parameters which imply zero-crossing timescales from several years to several decades.

**Figure 7.**
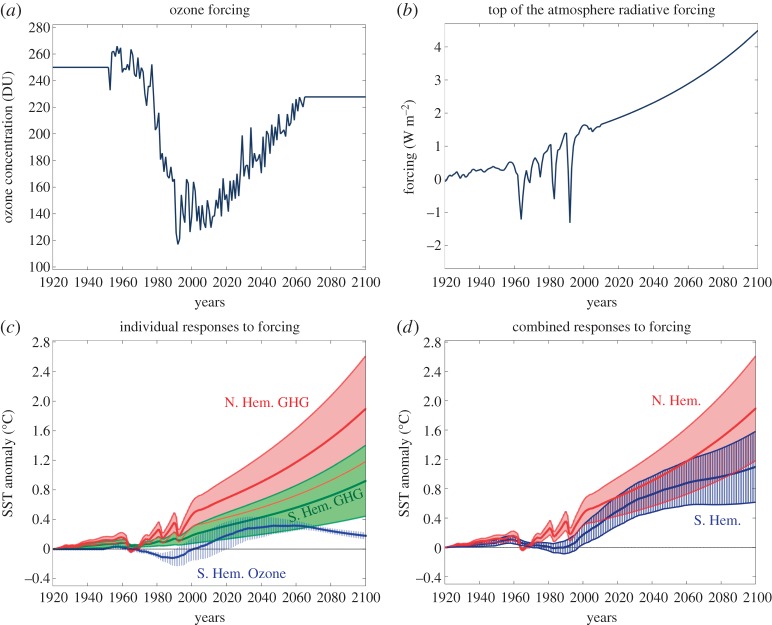
(*a*) Observed ozone concentration (in DU) over the Antarctic and projections into the future assuming that the ozone hole heals at the present observed rate (courtesy of WACCM and Diane Ivy, MIT). (*b*) Historical net TOA radiative forcing (in W m^−2^; dominated by GHGs) from Hansen *et al*. [60] and projections into the future assuming that the forcing increases smoothly to 4.5 W m^−2^ from 2010 to 2100, consistent with a standard RCP4.5 scenario. (*c*) Individual convolutions of the GHG and the ozone-hole forcing plotted on the top, with the respective GHG and ozone-hole CRFs plotted in figure 3, yielding estimates and projections of SST anomalies north of 50° N (Arctic: red due to GHGs) and between 50° S and 70° S (Antarctic: green due to GHGs and blue due to ozone-hole forcing). (*d*) Combined SST responses to GHG and ozone forcing north of 50° N (Arctic: red) and 50° *S* and 70° S (Antarctic: blue, sum of green and blue in (*c*).).

Our assumed GHG and ozone-hole forcing functions are shown in figure 7*a*,*b*. Their convolutions with the CRFs in figure 3 yield the SST time series plotted in figure 7*c*,*d*. The adjusted GHG forcing function is familiar and available from GISS (see [72] for a discussion). The total forcing trend is dominated by GHGs, but modified by volcanoes and anthropogenic aerosols. Note the downward spikes in the historical period due to volcanic activity. Projections in the future assume that the forcing increases smoothly to 4.5 W m^−2^ by 2100. We also assume that the same CRFs as calculated from abrupt CO_2_ forcing apply to the twentieth and twenty-first century forcing. This is indeed a rough approximation, since forcings other than CO_2_ (e.g. tropospheric aerosols, black carbon, volcanic aerosols) probably affect the Arctic differently than the Antarctic. Specifically, we are ignoring the ‘efficacy’ of individual climate forcings [72], and assuming all drive a similar response as that due to CO_2_. When interpreting the ozone CRF, we have assumed a linear scaling between the abrupt SAM wind-stress perturbation applied at the ocean surface (figure 5*a*) and an equivalent ozone step forcing *F*_step_∼100 DU.

The SST evolution is plotted in figure 7*c*,*d* and shows the effect of individual (left) and combined (right) forcings over both poles. The curves clearly reveal the differing responses to GHGs with the Arctic warming up more than twice as rapidly as the Antarctic: by 2050 the Arctic signal exceeds 1°C compared to the Antarctic rise of 0.4°C or so. The family of SST response curves to Antarctic ozone-hole forcing results in a cooling of order 0.1°C between 1980 and 2000 or so (but note the large spread due to the uncertainty in the form of the ozone CRF). From roughly 2000 onwards, however, the ozone-induced response begins to add to the warming induced by GHGs. The sum of the GHG and ozone-hole responses delays the warming trend by perhaps 20–30 years. It is tempting to suggest that this is the period through which we are now passing. By mid-century, however, the Antarctic ozone hole unequivocally adds to GHG warming but its contribution diminishes in the latter half of the century as the ozone hole heals.

## 5. Conclusion

We have presented a framework in which to consider the asymmetric response of the Arctic and Antarctic to GHG and ozone-hole forcing. The centrepiece of the framework are the respective GHG and ozone-hole CRFs which quantify in a suitably integral sense the transient response of the climate to ‘step’ changes in anthropogenic forcing.

GHG CRFs and linear response theory are known to be a useful tool for representing the global response to anthropogenic forcing [60]. Here, we have applied the approach regionally rather than globally, thus enabling us to contrast Arctic and Antarctic responses. The central role of the ocean circulation in setting the SST response to GHG forcing is illustrated by comparing figure 2*a* to *b*. Clearly, an ocean-only model can capture the broad spatial patterns and timing of the response. Delayed (accelerated) warming in the Antarctic (Arctic) is a consequence of anomalous advection of heat out of (into) the region by the ocean.

Ozone CRFs have only just recently been computed and in very few models. The first preliminary experiments with a highly idealized coupled model and a very sophisticated one are described in [39]. The two models yield quite different timescales for the onset of the slow warming processes. It would be of great utility if other coupled modelling groups carried out similar CRF calculations for an Antarctic ozone hole in which an ‘impulse’ ozone hole forcing with a repeating seasonal cycle is used to perturb the coupled atmosphere, ocean, ice system. This would expose the elemental processes, patterns and timescales at work and the differences across models. Indeed perhaps the biggest uncertainty is in the response of the surface climate around Antarctica to ozone-hole forcing. In the context of our framework, this involves understanding and quantifying the form of the ozone-hole CRF, figure 3*b*. Here, we have considered a range of parameters (table 1) and described the CRF in terms of a simple analytical expression, equation (4.2). Further research is required to understand what processes control its shape, whether it is well represented in models, and how we might constrain its form from observations.

Once the CRFs are quantified, we can use them to consider what might, or might not happen, for plausible anthropogenic forcing functions, as in figure 7. It is tempting to suggest that the current slight cooling of the climate around Antarctica might be a consequence of the cooling effects of the ozone hole which peaked around the turn of the century, offsetting the delayed warming tendencies of GHGs, but as the century proceeds GHG and ozone-hole forcing are likely to both contribute to warming around Antarctica. However, as we have seen, such warming effects are mitigated by advection of heat by ocean circulation away from Antarctica. The opposite happens over the Arctic where warming is accelerated by ocean heat transport across the Arctic circle. Finally, an important unresolved question is the extent to which natural variability confounds attempts to rationalize the problem. Perhaps nature is following one ensemble member of a plethora of other, equally plausible/possible trajectories.

## Funding statement

J.M. and Y.K. would like to acknowledge support from the NASA MAP program and UH from the NSF FESD program. K.A. was supported by a James S. McDonnell Foundation Postdoctoral Fellowship. J.S. received support from the Joint Program on the Science and Policy of Global Change, which is funded by a number of federal agencies and a consortium of 40 industrial and foundation sponsors, and from NSF grant #1259388. The authors are grateful to G. Forget for help with the model configuration used in this study, a simplified version of a fully global setup developed by the ‘Estimating the Circulation and Climate of the Ocean’ (ECCO) project built around the MITgcm.

## Footnotes

1 In figure 3, we define the Arctic as polewards of 50° N and the Antarctic as the band between 50° S and 70° S. We choose SSTs because here we focus on the role of the ocean.

2 It is important to note that GHGs also project on to surface westerlies. Such effects are implicit in the CRF's obtained from coupled models shown in figure 3*a*.
